# Effectiveness and safety of dual therapy with rilpivirine and boosted darunavir in treatment-experienced patients with advanced HIV infection: a preliminary 24 week analysis (RIDAR study)

**DOI:** 10.1186/s12879-019-3817-6

**Published:** 2019-02-28

**Authors:** Juan Pasquau, Samantha E. de Jesus, Piedad Arazo, María J. Crusells, María J. Ríos, Fernando Lozano, Javier de la Torre, María J. Galindo, Jorge Carmena, Jesús Santos, Carlos Tornero, Guillermo Verdejo, Gloria Samperiz, Zaira Palacios, Carmen Hidalgo-Tenorio, on behalf of the RIDAR Study Group, Juan Pasquau, Samantha E. de Jesus, Piedad Arazo, María J. Crusells, María J. Ríos, Fernando Lozano, Javier de la Torre, María J. Galindo, Jorge Carmena, Jesús Santos, Carlos Tornero, Guillermo Verdejo, Gloria Samperiz, Zaira Palacios, Carmen Hidalgo-Tenorio, Carlos Dueñas, Jose A. Terron, Miguel García-Deltoro, María T. Rubio, Miguel A. Cárdenes

**Affiliations:** 1 Hospital Universitario Virgen de las Nieves, Granada, Spain; 2 Hospital Miguel Servet, Zaragoza, Spain; 3 Hospital Lozano Blesa, Zaragoza, Spain; 4 Hospital Virgen Macarena, Sevilla, Spain; 5 Hospital de Valme, Sevilla, Spain; 6 Hospital Costa del Sol, Marbella, Spain; 7 Hospital General Universitario de Valencia, Valencia, Spain; 8 Hospital Universitario Doctor Peset, Valencia, Spain; 9 Hospital Virgen de la Victoria, Málaga, Spain; 10 Hospital Francesc de Borja, Gandía, Spain

**Keywords:** HIV, Simplification, Dual therapy, Rilpivirine, Darunavir, Nuke-sparing regimens

## Abstract

**Background:**

The objective was to analyze the effectiveness and safety of dual therapy with rilpivirine plus boosted-darunavir (RPV + bDRV) in real-life patients.

**Methods:**

Observational, retrospective, multi-center study in HIV+ patients who had received RPV + bDRV for 24 weeks to optimize/simplify their previous antiretroviral treatment. We determined the percentage of patients without virologic failure (2 consecutive viral loads > 50 copies/mL) at 24 weeks of treatment.

**Results:**

The study included 161 patients from 15 hospitals with median age of 49 years; 29.3% had previous AIDS stage and median CD4+ lymphocyte nadir of 170 cells/uL. They had been diagnosed with HIV for a median of 17 years and had received 14 years of ART, with five previous treatment combinations, and 36.6% had a history of virological failure. The reasons for the switch were simplification/optimization (49.7%), toxicity/intolerance (17.4%), or inadequate effectiveness of previous ART (10.6%).

Baseline VL of 50–1000 copies/mL was recorded in 25.5% of the patients. In the“*intention-to-treat*” analysis at 24 weeks, 87.6**%** of 161 patients continued the study treatment without virologic failure criteria.

In the “*on treatment*” analysis (excluding patients who discontinued treatment with dual therapy for any reason other than virologic failure) the efficacy was **94.6%** (141/149 patients).

**Conclusions:**

Dual therapy with RPV + DRVb proved to be effective and safe in patients with advanced HIV infection, long exposure to ART, low CD4 nadir, previous virologic failure, and/or history of ineffective ART.

## Background

The life expectancy of HIV-infected individuals is approaching that of the general population due to the effectiveness and good tolerability of new antiretroviral drugs.

The main concerns over antiretroviral therapy (ART) are no longer efficacy and tolerance, but antiretroviral drug-related toxicity [1], especially over the long term. This toxicity tends to be subclinical and cumulative and has the potential to interact with comorbidities, aging, and other processes related to immune activation and inflammation in HIV infection. It is mainly associated with Nucleoside Analogue.

Reverse Transcriptase Inhibitors (NRTI) [2–8], whose frequent use in pairs in classic Triple Therapy (TT) exerts a synergic antiviral effect but also increases the potential toxicity.

For this reason, nuke-sparing regimens (NSRs), that do not include one or both NRTIs, have been developed to reduce and prevent ART-related toxicity [9]. The development of novel and highly effective antiretroviral (ARV) drugs with a high genetic barrier against the development of drug resistance, such as protease inhibitors (PI) [10, 11], allows treatments to be simplified, with the administration of fewer drugs. Permanent TT may no longer be necessary and safe simplification strategies are now available to simplify ART [12].

Numerous studies (clinical trials, observational studies, and meta-analyses) have found that the efficacy of PI monotherapy (MT) [13–27] and dual therapy (DT) [28–31] with lamivudine (3TC) and boosted PIs (generally in patients without advanced HIV infection who had received suppressive ART for at least 6–12 months and/or had no history of virologic failure [VF]) was not inferior to that of TT [15, 17, 18, 20, 23, 24, 28–34]. Both of these simplification strategies maintain viral suppression while avoiding the development of resistance mutations [13–27, 35] and controlling immune activation and chronic inflammation [36–41], even in HIV sanctuaries and biological reservoirs [42–48], similarly to TT and with a good cost-effectiveness ratio [12, 49–54].

Fewer data are available on the application of DT in patients with a less favorable profile (advanced HIV infection, long exposure to ART, history of virological failures, long-term toxicity), although they have generally proven high efficacy and a reduction of possible toxicity [55–62].

DT with rilpivirine and boosted darunavir (RPV + bDRV) is an attractive NSR that appears to combine both a high efficacy and genetic barrier with a lower pill burden, good tolerance and toxicity profile. However, despite its utilization in clinical practice, there has been little research on the outcomes.

With this background, we performed a retrospective multi-center investigation to analyze the profile of patients prescribed with this combination and study its efficacy and safety. Confirmation that these NSRs are safe and effective will help to consolidate them as an optimized alternative for ART that could improve the long-term prognosis of HIV-infected patients and reduce treatment costs.

## Methods

### Study design

An observational, retrospective, multi-center study was conducted in HIV-infected patients who switched to DT with rilpivirine (RPV) (25 mg, once daily) and boosted darunavir (bDRV) (800 mg, once daily) (with either ritonavir or cobicistat).

### Objectives

The main objective of the study was to analyze the effectiveness of the DT by calculating the proportion of patients with virologic success (defined as 24 weeks of follow up without VF, considering VF as two consecutive RNA HIV-1 > 50 copies/mL).

Secondary objectives were to establish: a) proportion of patients with RNA HIV-1 < 50 copies/mL at week 24 of treatment; b) stratification of all viral load determinations obtained during exposure to the DT; c) Incidence of new adverse events; d) Impact of the DT on lymphocyte subpopulations, lipid profile, and liver and kidney function; e) Analysis of possible differences in efficacy and potential toxicity between darunavir/ritonavir (DRV/r) and darunvir/cobicistat (DRV/c).

### Inclusion criteria and variables

Patients from 15 Spanish hospitals were evaluated, with the following inclusion criteria: infection with HIV-1, age > 18 years, and receipt of antiretroviral treatment with RPV + bDRV before December 31st 2015 with a minimum follow-up period of 24 weeks. They were also required to have a baseline viremia at switch (bVL) < 1000 copies/mL and to have signed informed consent to retrieve data from their medical records.

A standardized electronic database was use to collect the following variables: age, gender, date of HIV diagnosis, VL at diagnosis, CD4 lymphocyte cell count at diagnosis, CD4 nadir, HIV stage (CDC), date of first ART, number of previous ART combinations, previous ART, previous VF (and ART received and genotype mutations at time of VF), reason for switch to study combination (toxicity, intolerance, VF, simplification/optimization or other reasons), booster (ritonavir or cobicistat), weeks of exposure to ritonavir and cobicistat, and HIV- 1 RNA, CD4 cell count, and bloodwork at baseline, 4–8 weeks, 9–23 weeks, and ≥ 24 weeks.

New adverse events during exposure to DT and reasons for not completing 24 weeks of follow-up were also collected (toxicity/intolerance, VF, voluntary drop-out, or others).

### Statistical analysis

For the first and secondary objectives of virologic efficacy, an intention-to-treat (ITT) analysis was carried out (considering losses as failures) as well as an on-treatment (OT) analysis, excluding patients who discontinued treatment with the DT for any reason other than VF (voluntary discontinuation of treatment, toxicity/intolerance, medical decision, patient’s decision).

Means with standard deviation, medians, and interquartile ranges were calculated for quantitative variables and absolute and relative frequencies for qualitative variables. After applying the Kolgomorov-Smirnov test to check the distribution of variables, effects of the DT on change in analytical parameters (lipids, liver and kidney function) were analyzed using the paired samples t-test when the distribution was normal or the signed-rank Wilcoxon test when it was not. For bivariate analyses, the Student’s t-test was applied when the distribution was normal and the Mann-Whitney test when it was not. SPSS v20.0 (IBM Corp, Armonk, NY) was used for data analyses, and the significance level was 0.05 for all tests.

### Ethical aspects

The study was approved by the regional ethics committee of Andalusia (CCEIBA) on April 25, 2016 and participants provided written informed consent for data from their medical records to be collected.

## Results

### General description of study population

One hundred and eighty-nine patients switched to RPV + bDRV: 28 did not meet inclusion criteria (20 with baseline VL > 1000 copies/mL and 8 with follow-up of < 24 weeks). Therefore, the final study sample comprised 161 patients with median age of 49 years (IQR 44–53), median time since HIV diagnosis of 17 years (IQR 10–23), receipt of ART for median of 14 years (IQR 6–18), and median of 5 previous ART combinations (IQR 3–7); 29.3% had a history of AIDS (stage C or 3 according to CDC), and the median CD4 lymphocyte nadir was 170 cells/μL (IQR 73–290) [Table 1]. ART regimens before the switch to DT included NRTI in 59% of patients, non-nucleoside reverse transcriptase inhibitors (NNRTIs) in 56.5%, protease inhibitors (PI) in 79.5%, and integrase inhibitors (II) in 9.9%; TT had been received by 57.1% of the patients, DT by 32.9%, and MT by 9.9%. Of the patients on MT, the reason for switch was optimization in 43.8% patients and VF in 43.8%; bVL was < 50 copies/mL in 6.3%.

**Table 1:** General description of study population

	*N* = 161
Age in years, median (IQR)	49 (44–53)
Male sex, n (%)	126 (78.3)
Years since HIV diagnosis, median (IQR)	17 (10–23)
CD4 nadir, median (IQR)	170 (73–290)
History of AIDS, n (%)	47 (29.3)
N° previous ART combinations, median (IQR)	5 (3–7)
Years on ART, median (IQR)	14 (6–18)
Previous ART that included, n (%):	
NRTI	95 (59)
NNRTI	91 (56.5)
PI	128 (79.5)
II	16 (9.9)
ART strategy, n(%):	
Triple therapy	92 (57.1)
Dual therapy	53 (32.9)
Monotherapy	16 (9.9)
Baseline VL (bVL):	
< 50 copies/mL, n (%)	120 (74.5)
50–1000 copies/mL, n (%)	41 (25.5)
Baseline CD4 count, median (IQR)	618 (370–861)

*IQR***:** interquartile range; *ART*: antiretroviral therapy; *NRTI*: nucleoside analog reversetranscriptase inhibitors; *NNRTI*: non-nucleoside analog reverse-transcriptase inhibitors; *PI*: protease inhibitors; *II*: integrase inhibitors; *VL*: viral load

At baseline, 25.5% did not have suppressed HIV viremia (baseline viral load of 50–1000 copies/mL), although the baseline CD4 lymphocyte cell count was 618 cells/μL (IQR 370–861) [Table 1]; 36.6% (59/161) had previous VF and data were available on the drug therapy at the time of the VF in 51 of these, being PI in 90.2% (46/51) and 9.3% of patients had previous virological failure while receiving NNRTI. In 58.8% (30/51) of cases, the PI was DRV. Hence, one-third of the patients in this study had experienced previous VF while receiving PI and one-fifth (21.5%) while receiving DRV.

The main reason for the switch to a DT with RPV + bDRV was simplification/optimization of their ART (49.7%), followed by previous ART toxicity (17.4%), and insufficient effectiveness of previous ART (10.6%).

Initially, the DT boosted with ritonavir (DRV/r) was administered to 131 patients (81.4%), 29 of whom switched to cobicistat (DRV/c) during the first 24 weeks of treatment. A total of 59 patients (36.6%) were exposed to DRV/c during the study period.

### Effectiveness of RPV + bDRV at 24 weeks of treatment

**Fig. 1:**
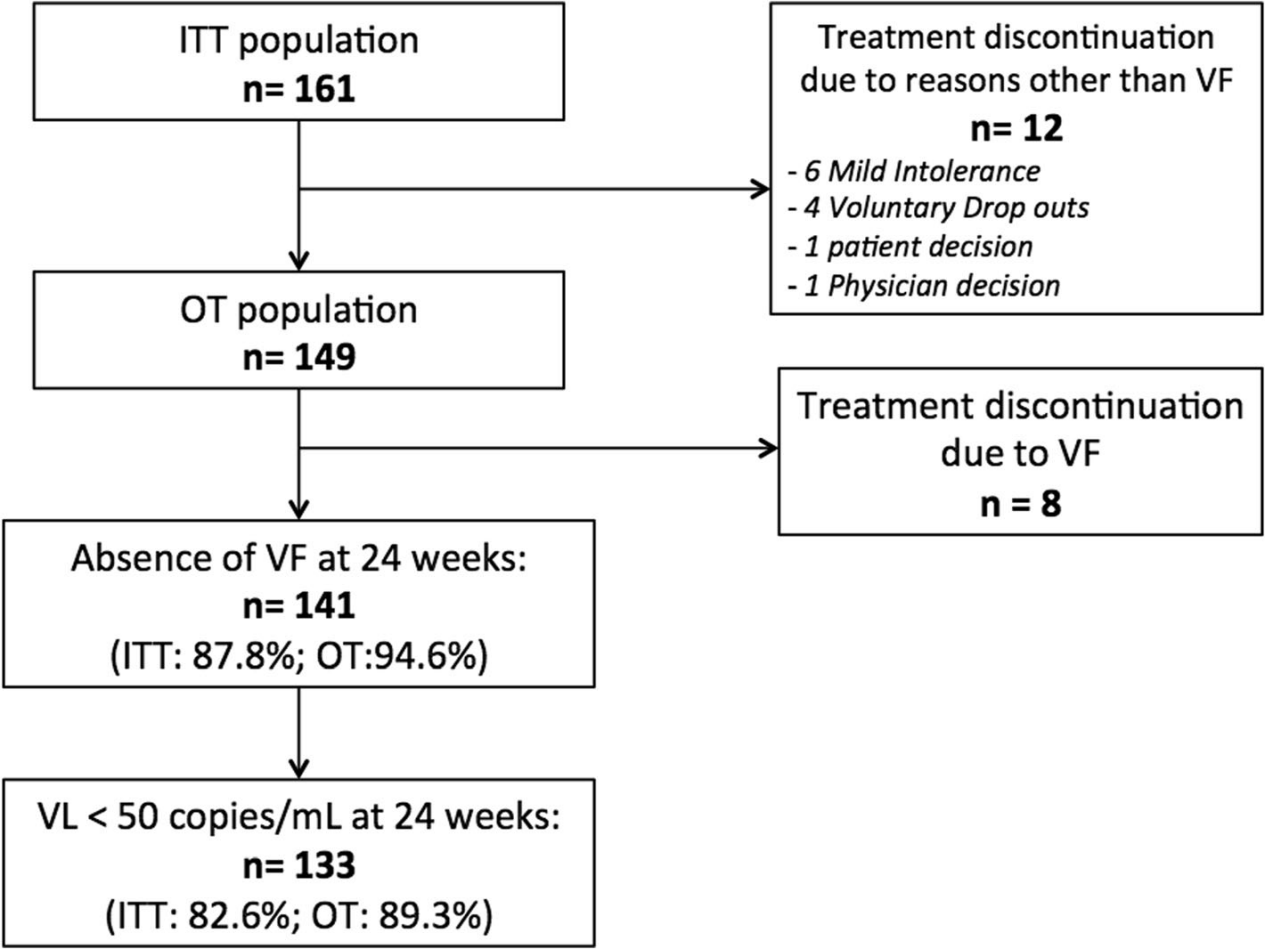
Flow chart of study patients

Figure 1 depicts the flow chart of patients through the study. The ITT population included all 161 patients, with a median follow up of 33 weeks. At week 24, 87.6% (141/161) of patients continued receiving RPV + bDRV and showed no VF criteria, while 17 patients discontinued this DT: 6 for mild toxicity/intolerance, 4 due to voluntary abandonment of ART, 1 by physician decision (due to one VL > 50 copies/mL), 1 by patient decision to return to previous ART and 8 VF (3 confirmed after week 24).

Excluding the 12 patients who dropped out of the study for reasons other than VF (population for OT analysis: 149 patients), 94.6% (141/149) completed the study without showing VF criteria.

In 82.6% (133/161) of the study population, the VL was < 50 copies/mL at 24 weeks (*ITT analysis).* In 89.3% (133/149) of the OT population, the VL was < 50 copies/mL at 24 weeks (*OT analysis*).

As shown in Table 2, genotyping was performed in three of the total of eight patients with VF and no drug resistance mutations were detected, while two cases were associated with poor adherence to treatment. Resuppression of HIV viremia was achieved in all eight cases with no modification of the DT (3 cases) or after switching to a TT (4 cases) or different DT (1 case).

**Table 2:** Virologic failures with RPV + bDRV

Patient	Baseline VL	Previous ART	VL at time of VF	Observations
1	33	ABC/3TC + DRV/r	81–393	Resuppression without switch (continued on RPV + bDRV) No drug resistance mutations.
2	159	ETV + DRV/r	67–96	Switch to cART (to include omeprazol). No genotyping.
3	759	ETV + DRV/r	560–7530	Switch to RPV+ bDRV + DTG No genotyping.
4	210	ETV + DRV/r	191–131	Resuppression without switch (continued on RPV + bDRV) No genotyping.
5	< 50	ETV + DRV/r	16,500 (390 five days later)	Poor adherence No switch (continued on RPV + bDRV) No genotyping.
6	< 50	ETV + RAL + DRV/r	270–1197	Switch to ETV + DRV/r (post-switch VL of 23 copies) No drug resistance mutations
7	< 50	ETV + RAL + ATV/r	220–612	Switch to ABC/3TC + DRV/r No drug resistance mutations
8	95	TDF/FTC + NVP	173,000-1590	Poor adherence No genotyping

*VL***:** viral load; *ART***:** antiretroviral therapy; *VF***:** virological failure; *ABC:* abacavir; 3TC:

lamivudine; *DRV*: darunavir; *bDRV*: boosted darunavir; *ETV*: etravirine; *RAL*: raltegravir; *RPV:* rilpivirine; *TDF*: tenofovir; *ATV*: atazanavir; *NVP*: nevirapine; *DTG*:

dolutegravir

Out of the 334 VL determinations during exposure to RPV + bDRV, over 105 patients-years, < 50 copies/mL were found in 87.7% (< 20 copies/mL in 79%), 50–1000 copies/mL in 11.1%, and > 1000 copies/mL in 1.2%; 237 of these determinations were obtained during exposure to RPV + DRV/r, with 87.3% being < 50 copies/mL, and 97 during exposure to RPV + DRV/c, with 88.7% being < 50 copies/mL.

The last observed VL determination of the 159 patients with virologic data after the switch was < 50 copies/mL in 86.2% of patients (< 20 copies/mL in 80.5%), between 50 and 1000 copies/mL in 12.6%, and > 1000 copies/mL in 1.3%.

During the 24 weeks of exposure to the DT, the CD4 lymphocyte cell count increased by 34 cells/mm3 from 630 cells/mL to 667 cells/mm3 (*p* = 0.071) and the CD4/CD8 ratio by 0.04 from 0.75 to 0.79 (*p* = 0.004).

Patients with previous failure while receiving darunavir were not more likely to present VF with the study DT [13.3% (4/30) vs 5.5% (1/18), *p* = 0.348] and overall baseline viral load > 50 copies/mL was not a predictor of VF (9.8% vs 3.3%, *p* = 0.115).

We analyzed the influence of CD4 nadir on VF and found no statistically significant differences between those who failed and those who didn’t (257 vs 156 cells/mm3, *p* = 0.470). There were also no differences in VF rate between those who received the DT boosted with ritonavir vs cobicistat (7,5% vs 7,1%, *p* = 1.000).

### Safety of RPV + bDRV

Although no severe adverse events were notified, six patients switched therapies due to mild toxicity or intolerance (Table 3).

**Table 3:** Adverse events during the study

	N (%)
Mild adverse events	6 (3.7)
Anxiety and hypercholesterolemia	1 (0.6)
Abdominal pain, dyspepsia, and asthenia	1 (0.6)
Irascibility and insomnia	1 (0.6)
Pyrosis	1 (0.6)
Sexual dysfunction	1 (0.6)
Diarrhea	1 (0.6)
Grade 3–4 adverse events	0 (0)

### Impact of RPV + bDRV on analytical parameters (lipid profiles, and kidney and liver functions)

Statistically significant differences between values at baseline and 24 weeks were found in creatinine (0.944 mg/dL vs. 0.977 mg/dL, *p* = 0.001), total cholesterol (183 mg/dL vs. 196 mg/dL, *p* < 0.001), HDL-cholesterol (48 mg/dL vs. 52 mg/dL, *p* = 0.005), LDL-cholesterol (107 mg/dL vs. 124 mg/dL, *p* = 0.003), GOT (40.9 U/L vs. 30.8 U/L, *p* = 0.031), GPT (45.9 U/L vs. 30.3 U/L, *p* = 0.011), GGT (57.5 U/L vs. 59.4 U/L, *p* = 0.040), and alkaline phosphatase (98 U/L vs. 93 U/L, *p* = 0.027) but not in total cholesterol/HDLcholesterol ratio (Table 4). A higher increase of LDL-cholesterol was observed with ritonavir (+ 20 mg/dL) than with cobicistat (+ 10 mg/dL), but the difference did not reach statistical significance (*p* = 0.462).

**Table 4:** Impact of the dual therapy on analytical parameters

	Baseline	24 weeks	*P* value
CD4 lymphocytes (cells/uL)	630	667	0.071*
CD4/CD8 Ratio	0.749	0.788	**0.004**
Creatinine (mg/dL)	0.944	0.977	**0.001**
Glucose (mg/dL)	95.2	96.5	0.057
Total Cholesterol/HDL	4,13	4,10	0.107
Total Cholesterol (mg/dL)	183	196	< 0.001*
HDL-cholesterol (mg/dL)	48	52	**0.005**
LDL-cholesterol (mg/dL)	107	124	0.003*
Triglycerides (mg/dL)	144	157	0.172
Bilirubin (mg/dL)	0.77	0.64	0.827
GOT (U/L)	40.9	30.8	**0.031**
GPT (U/L)	45.9	30.3	**0.011**
GGT (U/L)	57.5	59.4	**0.040**
Alkaline Phosphatase (U/L)	98	93	**0.026**
Hemoglobin (g/dL)	15	14.8	**0.009**
Platelets (×10^3/μL)	190	189	0.346

*paired samples t-test (otherwise with Wilcoxon signed-rank test)

## Discussion

Data obtained in this study confirm that nuke-sparing DT with RPV + bDRV may be an acceptable alternative to triple therapy in suppressed and stable HIV-infected patients, as previously suggested in the PROBE CT [63]. However, the present patients generally had a long history of exposure to HIV and ART and included numerous cases of severe previous immunodepression (AIDS stage and/or low CD4 nadir), VF, toxicity associated with antiretroviral drugs, and even a previous non suppressive ART. These conditions have usually been considered as exclusion criteria in studies of MT and DT with 3TC. Nevertheless, the DT under study was found to achieve and maintain viral suppression in > 90% of the present patient population.

Despite the disadvantageous profile of our study population, the proportion of virologic suppression obtained with this DT was similar to that obtained with TT (including stable patients with no history of VF and with suppressed viremia in this switch scenario). Stratification of all viral load determinations in the entire cohort during the study period showed similar rates of blips and VFs, and almost all of the latter could be attributed to poor treatment adherence**.** No drug resistance mutations against the protease or the inverse transcriptase were observed in any case, and all patients achieved viral resuppression by maintaining the DT or adding a third drug. Although RPV has a low genetic barrier and patients who showed VF could potentially develop resistance to the drug, in this study there were no VF with real exposure to the DT (multiple patients reported poor treatment adherence) and/or high viral loads and few drug resistance tests were performed.

This is a preliminary analysis of a cohort that we are still following, but we believe that a ‘24 weeks Analysis’, considering a threshold of 50 copies/mL for VF, is enough to determine virologic effectiveness for previously suppressed patients. This is the minimum timeframe required to prove ART’s ability to suppress viral replication in naïve patients [64–68] and for rescue strategies in patients with prior virological failure [68]. Twenty-four weeks is also the minimum timeframe required in switch studies to consider a previous HAART stable and effective [69, 70], and we know that the maximum suppression of HIV viremia can be achieved with < 20 weeks of treatment [71], that virological failures with simplification strategies occur during the first months [72] and that this rate does not increase with follow-up time [73].

Despite their long history of ART, the immunological recovery was similar to that reported for TT, with an increase in CD4 lymphocytes and CD4/CD8 ratio.

Tolerance of the combination was generally good, although several patients asked to switch to previous or alternative treatments due to toxicity, which was considered mild in all of these cases.

A slight increase in total cholesterol and LDL-cholesterol levels was observed with the DT under study; however, there was also an increase in HDL-cholesterol levels, with no change in the atherogenic index over the 24-week observation period. There was a significant decrease in transaminase levels, implying a reduction in the potential toxicity of this DT, which supports the idea that NRTIs such as tenofovir could have certain level of hepatotoxicity.

More than one-third of the patients received DRV/c (from the start of the study in half of these cases) and showed no difference in safety and effectiveness outcomes with those receiving DRV/r.

Study limitations include its retrospective, multi-center design, although the necessary data were recovered for almost all patients. Inclusion bias was minimized by recruiting all patients who had been prescribed with the DT under study in the participating hospitals. However, it was not possible to evaluate potential long-term changes in the toxicity of this combination due to the study design and short follow-up period.

## Conclusions

Dual therapy with RPV + bDRV in the clinical setting has proven to be effective, even in patients with advanced HIV infection, extended exposure to ART, low CD4 lymphocyte nadir, history of VF, and/or previous non-suppressive ART.

## Abbreviations

ART: Antiretroviral therapy
ARV: Antiretroviral
bDRV: Boosted darunavir
bVL: Baseline viral load
DRV: Darunavir
DRV/c: Darunavir/cobicistat
DRV/r: Darunavir/ritonavir
DT: Dual therapy
ITT: Intention to treat analysis
MT: Monotherapy
NRTI: Nucleoside analogue reverse transcriptase inhibitors
NSR: Nuke-sparing regimen
OT: On treatment analysis
PI: Protease inhibitor
RPV: Rilpivirine
TT: Triple therapy
VF: Virological failure
VL: Viral load

## References

[1] Margolis AM, Heverling H, Pham PA, Stolbach A (2014). A review of the toxicity of HIV medications. J Med Toxicol.

[2] Friis-Møller N, Ryom L, Smith C, Weber R, Reiss P, Dabis F (2016). An updated prediction model of the global risk of cardiovascular disease in HIV-positive persons: the data-collection on adverse effects of anti-HIV drugs (D:a:D) study. Eur J Prev Cardiol.

[3] Falcinelli E, Francisci D, Belfiori B, Petito E, Guglielmini G, Malincarne L (2013). In vivo platelet activation and platelet hyperreactivity in abacavir-treated HIV-infected patients. Thromb Haemost.

[4] Scherzer R, Estrella M, Li Y, Choi AI, Deeks SG, Grunfeld C (2012). Association of tenofovir exposure with kidney disease risk in HIV infection. AIDS..

[5] Bedimo R, Maalouf NM, Zhang S, Drechsler H, Tebas P (2012). Osteoporotic fracture risk associated with cumulative exposure to tenofovir and other antiretroviral agents. AIDS..

[6] Mulligan K, Glidden DV, Anderson PL, Liu A, McMahan V, Gonzales P (2015). Effects of Emtricitabine/Tenofovir on bone mineral density in HIV-negative persons in a randomized, double-blind. Placebo-Controlled Trial Clin Infect Dis.

[7] Leeansyah E, Cameron PU, Solomon A, Tennakoon S, Velayudham P, Gouillou M (2013). Inhibition of telomerase activity by human immunodeficiency virus (HIV) nucleos(t) ide reverse transcriptase inhibitors: a potential factor contributing to HIV-associated accelerated aging. J Infect Dis.

[8] Kovari H, Sabin CA, Ledergerber B, Ryom L, Reiss P, Law M, et al. Antiretroviral Drugs and Risk of Chronic Alanine Aminotransferase Elevation in Human Immunodeficiency Virus (HIV)-Monoinfected Persons: The Data Collection on Adverse Events of Anti-HIV Drugs Study. Open Forum Infect Dis. 2016;3(1):ofw009. 10.1093/ofid/ofw009 PMC4767274 26925429

[9] Carrero-Gras A, Antela A, Muñoz-Rodríguez J, Díaz-Menéndez M, Viciana P, Torrella-Domingo A (2014). Nuke-sparing regimens as a main simplification strategy and high level of toxicity resolution after antiretroviral switch: the SWITCHART study. J Int AIDS Soc.

[10] Rabi SA, Laird GM, Durand CM, Laskey S, Shan L, Bailey JR (2013). Multi-step inhibition explains HIV-1 protease inhibitor pharmacodynamics and resistance. J Clin Invest.

[11] Lathouwers E, Gupta S, Haddad M, Paquet A, de Meyer S, Baugh B (2015). Trends in darunavir resistance-associated mutations and phenotypic resistance in commercially tested United States clinical samples between 2006 and 2012. AIDS Res Hum Retrovir.

[12] Pasquau J, Hidalgo-Tenorio C (2015). Nuke-sparing regimens for the long-term care of HIV infection. AIDS Rev.

[13] Arribas JR, Pulido F, Delgado R, Lorenzo A, Miralles P, Arranz A (2005). Lopinavir/ritonavir as single-drug therapy for maintenance of HIV-1 viral suppression: 48-week results of a randomized, controlled, open-label, proof-of-concept pilot clinical trial (OK study). J Acquir Immune Defic Syndr.

[14] Arribas JR, Delgado R, Arranz A, Muñoz R, Portilla J, Pasquau J (2009). Lopinavir-ritonavir monotherapy versus lopinavir-ritonavir and 2 nucleosides for maintenance therapy of HIV: 96-week analysis. J Acquir Immune Defic Syndr.

[15] Arribas JR, Horban A, Gerstoft J, Fätkenheuer G, Nelson M, Clumeck N (2010). The MONET trial: darunavir/ritonavir with or without nucleoside analogues, for patients with HIV RNA below 50 copies/ml. AIDS..

[16] Pasquau J, Hidalgo-Tenorio C, Vergara A, al e. A clinical trail to compare the quality of life of HIV+ patietns who start monotherapy with LPV/r versus continuing triple therapy with a boosted PI. HIV Drug Therapy Congress; Glasgow2012.

[17] Valantin MA, Lambert-Niclot S, Flandre P, Morand-Joubert L, Cabiè A, Meynard JL (2012). Long-term efficacy of darunavir/ritonavir monotherapy in patients with HIV-1 viral suppression: week 96 results from the MONOI ANRS 136 study. J Antimicrob Chemother.

[18] Antinori A, Clarke A, Svedhem-Johansson V, Arribas JR, Arenas-Pinto A, Fehr J (2015). Week 48 efficacy and central nervous system analysis of darunavir/ritonavir monotherapy versus darunavir/ritonavir with two nucleoside analogues. AIDS..

[19] Paton NI, Stöhr W, Arenas-Pinto A, Fisher M, Williams I, Johnson M (2015). Protease inhibitor monotherapy for long-term management of HIV infection: a randomised, controlled, open-label, non-inferiority trial. Lancet HIV..

[20] López-Cortés LF, Castaño MA, López-Ruz MA, Rios-Villegas MJ, Hernández-Quero J, Merino D (2016). Effectiveness of ritonavir-boosted protease inhibitor monotherapy in clinical practice even with previous Virological failures to protease inhibitor-based regimens. PLoS One.

[21] Pasquau J, López-Cortés L, Mayorga MI, Viciana P, Del Mar AM, Ríos MJ (2014). Monotherapy with darunavir/ritonavir is effective and safe in clinical practice. J Int AIDS Soc.

[22] Santos JR, Llibre JM, Berrio-Galan D, Bravo I, Miranda C, Pérez-Alvarez S (2015). Monotherapy with boosted PIs as an ART simplification strategy in clinical practice. J Antimicrob Chemother.

[23] Guiguet M, Ghosn J, Duvivier C, Meynard JL, Gras G, Partisani M (2012). Boosted protease inhibitor monotherapy as a maintenance strategy: an observational study. AIDS..

[24] Cozzi-Lepri A, Gianotti N, Antorini A, al e. ICONA Foundation Study and Mono PI/r database study cohorts. EACS; Barcelona2015.

[25] Pasquau J, Garcia-Vallecillos C, Cruces-Moreno M (2016). In monotherapy, darunavir/cobicistat demonstrates equivalence to darunavir/ritonavir, and in selected patients is as effective as bitherapies or triple therapies. International congress of drug therapy in HIV infection.

[26] Mathis S, Khanlari B, Pulido F, Schechter M, Negredo E, Nelson M (2011). Effectiveness of protease inhibitor monotherapy versus combination antiretroviral maintenance therapy: a meta-analysis. PLoS One.

[27] Arribas J, Girard PM, Paton N, Winston A, Marcelin AG, Elbirt D (2014). Efficacy of PI monotherapy versus triple therapy for 1964 patients in 10 randomised trials. J Int AIDS Soc.

[28] Arribas JR, Girard PM, Landman R, Pich J, Mallolas J, Martínez-Rebollar M (2015). Dual treatment with lopinavir-ritonavir plus lamivudine versus triple treatment with lopinavir-ritonavir plus lamivudine or emtricitabine and a second nucleos(t) ide reverse transcriptase inhibitor for maintenance of HIV-1 viral suppression (OLE): a randomised, open-label, non-inferiority trial. Lancet Infect Dis.

[29] Mondi A, Fabbiani M, Ciccarelli N, Colafigli M, D'Avino A, Borghetti A (2015). Efficacy and safety of treatment simplification to atazanavir/ritonavir + lamivudine in HIV-infected patients with virological suppression: 144 week follow-up of the AtLaS pilot study. J Antimicrob Chemother.

[30] Perez-Molina JA, Rubio R, Rivero A, Pasquau J, Suárez-Lozano I, Riera M (2015). Dual treatment with atazanavir-ritonavir plus lamivudine versus triple treatment with atazanavir-ritonavir plus two nucleos(t) ides in virologically stable patients with HIV-1 (SALT): 48 week results from a randomised, open-label, non-inferiority trial. Lancet Infect Dis.

[31] Pulido F, Ribera E, Kagarde M, et al. Non-inferiority of dual-therapy with darunavir/ritonavir plus 3TC vs triple therapy with darunavir/ritonavir plus TDF/FTC or ABC/3TC for maintence of viral suppression: 48-week results of the DUAL-GESIDA 8014-RIS-EST45 trial. International congress of drug therapy in HIV infection; Glasgow: J Int AIDS Soc; 2016.

[32] Pulido F, Pérez-Valero I, Delgado R, Arranz A, Pasquau J, Portilla J (2009). Risk factors for loss of virological suppression in patients receiving lopinavir/ritonavir monotherapy for maintenance of HIV suppression. Antivir Ther.

[33] Stöhr W, Dunn DT, Arenas-Pinto A, Orkin C, Clarke A, Williams I (2016). Factors associated with virological rebound in HIV-infected patients receiving protease inhibitor monotherapy. AIDS..

[34] Gianotti N, Cozzi-Lepri A, Antinori A, Castagna A, De Luca A, Celesia BM (2017). Refining criteria for selecting candidates for a safe lopinavir/ritonavir or darunavir/ritonavir monotherapy in HIV-infected virologically suppressed patients. PLoS One.

[35] Pulido F, Arribas JR, Hill A, Van Delft Y, Moecklinghoff C (2011). Analysis of drug resistance during HIV RNA viraemia in the MONET trial of darunavir/ritonavir monotherapy. Antivir Ther.

[36] Arribas J, Hill A, Xi N, van Delft Y, Moecklinghoff C (2012). Interleukin-6 and C-reactive protein levels after 3 years of treatment with darunavir/ritonavir monotherapy or darunavir/ritonavir + two nucleoside reverse transcriptase inhibitors in the MONET trial. J Antimicrob Chemother.

[37] BenMarzouk-Hidalgo OJ, Torres-Cornejo A, Gutiérrez-Valencia A, Ruiz-Valderas R, Viciana P, López-Cortés LF (2015). Differential effects of viremia and microbial translocation on immune activation in HIV-infected patients throughout ritonavir-boosted darunavir monotherapy. Medicine (Baltimore).

[38] Estébanez M, Stella-Ascariz N, Mingorance J, Pérez-Valero I, Bernardino JI, Zamora FX (2014). Inflammatory, procoagulant markers and HIV residual viremia in patients receiving protease inhibitor monotherapy or triple drug therapy: a cross-sectional study. BMC Infect Dis.

[39] Chun TW, Murray D, Justement JS, Hallahan CW, Moir S, Kovacs C (2011). Relationship between residual plasma viremia and the size of HIV proviral DNA reservoirs in infected individuals receiving effective antiretroviral therapy. J Infect Dis.

[40] Eastburn A, Scherzer R, Zolopa AR, Benson C, Tracy R, Do T (2011). Association of low level viremia with inflammation and mortality in HIV-infected adults. PLoS One.

[41] Gandhi RT, McMahon DK, Bosch RJ, Lalama CM, Cyktor JC, Macatangay BJ (2017). Levels of HIV-1 persistence on antiretroviral therapy are not associated with markers of inflammation or activation. PLoS Pathog.

[42] Pérez-Valero I, González-Baeza A, Estébanez M, Monge S, Montes-Ramírez ML, Bayón C (2014). A prospective cohort study of neurocognitive function in aviremic HIV-infected patients treated with 1 or 3 antiretrovirals. Clin Infect Dis.

[43] Santos JR, Muñoz-Moreno JA, Moltó J, Prats A, Curran A, Domingo P (2013). Virological efficacy in cerebrospinal fluid and neurocognitive status in patients with long-term monotherapy based on lopinavir/ritonavir: an exploratory study. PLoS One.

[44] Torres-Cornejo A, BenMarzouk-Hidalgo OJ, Viciana P, Sánchez B, López-Ruz MA, López-Cortés LF, et al. Protease inhibitor monotherapy is effective in controlling human immunodeficiency virus 1 shedding in the male genital tract. Clin Microbiol Infect. 2016;22(1):98.e7-.e10. 10.1016/j.cmi.2015.09.028 26454060

[45] Lopez-Ruz MA, Navas P, López-Zúñiga MA, Gonzalvo MC, Sampedro A, Pasquau J (2016). Effect of monotherapy with Darunavir/ritonavir on viral load in seminal fluid, and quality parameters of semen in HIV-1-positive patients. PLoS One.

[46] Vinuesa D, Parra-Ruiz J, Chueca N, Alvarez M, Muñoz-Medina L, Garcia F (2014). Protease inhibitor monotherapy is not associated with increased viral replication in lymph nodes. AIDS..

[47] Pasquau Liaño J. Penetración en reservorios. En: Lopinavir potenciado con ritonavir en monoterapia para el tratamiento del virus de la inmunodeficiencia humana. Enf Infecc Microb Clin. 2008;26(Supl 16):41–6.

[48] Fletcher CV, Staskus K, Wietgrefe SW, Rothenberger M, Reilly C, Chipman JG (2014). Persistent HIV-1 replication is associated with lower antiretroviral drug concentrations in lymphatic tissues. Proc Natl Acad Sci U S A.

[49] Ciaffi L, Koulla-Siro S, Sawadogo A (2016). Dual therapy with a boosted protease inhibitor plus lamivudine is an effective maitenance strategy in patients on second-line antiretroviral therapy in Africa: the ANRS 12286/MOBIDIP trial. International congress of drug therapy in HIV infection.

[50] Baril JG, Angel JB, Gill MJ, Gathe J, Cahn P, van Wyk J (2016). Dual therapy treatment strategies for the Management of Patients Infected with HIV: a systematic review of current evidence in ARV-naive or ARV-experienced. Virologically Suppressed Patients PLoS One.

[51] Collins SE, Grant PM, Shafer RW (2016). Modifying antiretroviral therapy in Virologically suppressed HIV-1-infected patients. Drugs..

[52] Pasquau J, Gostkorzewicz J, Ledesma F, Anceau A, Hill A, Moecklinghoff C (2012). Budget impact analysis of switching to darunavir/ritonavir monotherapy for HIV-infected people in Spain. Appl Health Econ Health Policy.

[53] Oddershede L, Walker S, Paton N, Stöhr W, Dunn D, Sculpher M. Cost-effectiveness analysis of protease inhibitor monotherapy vs. ongoing triple-therapy in the long-term management of HIV patients. J Int AIDS Soc. 2014;17(4 Suppl 3):19498. 10.7448/IAS.17.4.19498 PMC4224943 25394007

[54] Ribera E, Martínez-Sesmero JM, Sánchez-Rubio J, Rubio R, Pasquau J, Poveda JL, et al. [Economic impact of optimising antiretroviral treatment in human immunodeficiency virus-infected adults with suppressed viral load in Spain, by implementing the grade A-1 evidence recommendations of the 2015 GESIDA/national AIDS plan]. Enferm Infecc Microbiol Clin 2017. 10.1016/j.eimc.2016.11.015 28109551

[55] van Lunzen J, Pozniak A, Gatell JM, Antinori A, Klauck I, Serrano O (2016). Brief report: switch to ritonavir-boosted Atazanavir plus Raltegravir in Virologically suppressed patients with HIV-1 infection: a randomized pilot study. J Acquir Immune Defic Syndr.

[56] Amin J, Boyd MA, Kumarasamy N, Moore CL, Losso MH, Nwizu CA (2015). Raltegravir non-inferior to nucleoside based regimens in second-line therapy with lopinavir/ritonavir over 96 weeks: a randomised open label study for the treatment of HIV-1 infection. PLoS One.

[57] Ruane PJ, Brinson C, Ramgopal M, Ryan R, Coate B, Cho M (2015). The Intelence aNd pRezista once a day study (INROADS): a multicentre, single-arm, open-label study of etravirine and darunavir/ritonavir as dual therapy in HIV-1-infected early treatment-experienced subjects. HIV Med..

[58] Gazzola L, Cicconi P, Ripamonti D, Di Filippo E, Gustinetti G, Di Biagio A (2014). Efficacy and safety of darunavir/ritonavir plus etravirine dual regimen in antiretroviral therapy-experienced patients: a multicenter clinical experience. HIV Clin Trials.

[59] Portilla J, Arazo P, Crusells J, Pérez-Martínez L, Martínez-Madrid O, Boix V, et al. Dual therapy with darunavir/r plus etravirine is safe and effective as switching therapy in antiretroviral experienced HIV-patients. The BITER Study. J Int AIDS Soc. 2014;17(4 Suppl 3):19803. 10.7448/IAS.17.4.19803 PMC4225347 25397547

[60] Calin R, Paris L, Simon A, Peytavin G, Wirden M, Schneider L (2012). Dual raltegravir/etravirine combination in virologically suppressed HIV-1-infected patients on antiretroviral therapy. Antivir Ther.

[61] Calza L, Magistrelli E, Colangeli V, Borderi M, Conti M, Mancini R (2016). Improvement in renal function and bone mineral density after a switch from tenofovir/emtricitabine plus ritonavir-boosted protease inhibitor to raltegravir plus nevirapine: a pilot study. Antivir Ther.

[62] Bernardino JI, Mocroft A, Mallon PW, Wallet C, Gerstoft J, Russell C (2015). Bone mineral density and inflammatory and bone biomarkers after darunavir-ritonavir combined with either raltegravir or tenofovir-emtricitabine in antiretroviral-naive adults with HIV-1: a substudy of the NEAT001/ANRS143 randomised trial. Lancet HIV..

[63] Maggiolo F, Di Filippo E, Valenti D, Serna Ortega PA, Callegaro A (2016). NRTI sparing therapy in Virologically controlled HIV-1 infected subjects: results of a controlled, randomized trial (Probe). J Acquir Immune Defic Syndr.

[64] Molina JM, Clotet B, van Lunzen J, Lazzarin A, Cavassini M, Henry K (2015). Once-daily dolutegravir versus darunavir plus ritonavir for treatment-naive adults with HIV-1 infection (FLAMINGO): 96 week results from a randomised, open-label, phase 3b study. Lancet HIV..

[65] Cevik M (2018). Orkin C.

[66] Kanters S, Socias ME, Paton NI, Vitoria M, Doherty M, Ayers D (2017). Comparative efficacy and safety of second-line antiretroviral therapy for treatment of HIV/AIDS: a systematic review and network meta-analysis. Lancet HIV.

[67] DHHS. Guidelines for the Use of Antiretroviral Agents in Adults and Adolescents Living with HIV 2018 [Available from: https://aidsinfo.nih.gov/guidelines.

[68] FDA. Human Immunodeficiency Virus-1 Infection: Developing Antiretroviral Drugs for Treatment [Available from: https://www.fda.gov/downloads/Drugs/GuidanceComplianceRegulatoryInformation/Guidances/UCM355128.pdf.

[69] Arribas JR, Clumeck N, Nelson M, Hill A, van Delft Y, Moecklinghoff C (2012). The MONET trial: week 144 analysis of the efficacy of darunavir/ritonavir (DRV/r) monotherapy versus DRV/r plus two nucleoside reverse transcriptase inhibitors, for patients with viral load < 50 HIV-1 RNA copies/mL at baseline. HIV Med.

[70] Perez-Molina JA, Rubio R, Rivero A, Pasquau J, Suárez-Lozano I, Riera M, et al. Simplification to dual therapy (atazanavir/ritonavir + lamivudine) versus standard triple therapy [atazanavir/ritonavir + two nucleos(t)ides] in virologically stable patients on antiretroviral therapy: 96 week results from an open-label, non-inferiority, randomized clinical trial (SALT study). J Antimicrob Chemother. 2016. 10.1093/jac/dkw379 27629070

[71] Ananworanich J, Eller LA, Pinyakorn S, Kroon E, Sriplenchan S, Fletcher JL (2017). Viral kinetics in untreated versus treated acute HIV infection in prospective cohort studies in Thailand. J Int AIDS Soc.

[72] Paton NI, Stöhr W, Oddershede L, Arenas-Pinto A, Walker S, Sculpher M (2016). The protease inhibitor monotherapy versus ongoing triple therapy (PIVOT) trial: a randomised controlled trial of a protease inhibitor monotherapy strategy for long-term management of human immunodeficiency virus infection. Health Technol Assess.

[73] Margolis DA, Gonzalez-Garcia J, Stellbrink HJ, Eron JJ, Yazdanpanah Y, Podzamczer D (2017). Long-acting intramuscular cabotegravir and rilpivirine in adults with HIV-1 infection (LATTE-2): 96-week results of a randomised, open-label, phase 2b, non-inferiority trial. Lancet..

